# Author Correction: Large-scale shift in the structure of a kelp forest ecosystem co-occurs with an epizootic and marine heatwave

**DOI:** 10.1038/s42003-021-01993-7

**Published:** 2021-04-06

**Authors:** Meredith L. McPherson, Dennis J. I. Finger, Henry F. Houskeeper, Tom W. Bell, Mark H. Carr, Laura Rogers-Bennett, Raphael M. Kudela

**Affiliations:** 1 Department of Ocean Sciences, University of California Santa Cruz, Santa Cruz, CA USA; 2 Department of Environmental Science, Policy, and Management, University of California Berkeley, Berkeley, CA USA; 3 Department of Geography, University of California Los Angeles, Los Angeles, CA USA; 4 Department of Applied Ocean Physics and Engineering, Woods Hole Oceanographic Institution, Woods Hole, MA USA; 5 Earth Research Institute, University of California Santa Barbara, Santa Barbara, CA USA; 6 Department of Ecology and Evolutionary Biology, University of California Santa Cruz, Santa Cruz, CA USA; 7 Coastal Marine Science Institute, Karen C. Drayer Wildlife Health Center, University of California Davis and California Department of Fish and Wildlife, Bodega Marine Laboratory, Bodega Bay, CA USA

**Keywords:** Community ecology, Ecology

Correction to: *Communications Biology* 10.1038/s42003-021-01827-6, published online 5 March 2021.

The original version of the Article contained an error in reference 32 in which the name of the first author, Eisaguirre, J. H., was omitted. The full correct reference is: Eisaguirre, J. H., Eisaguirre, J. M., Davis, K., Carlson, P. M., Gaines, S. D. & Caselle, J. E. Trophic redundancy and predator size class structure drive differences in kelp forest ecosystem dynamics. *Ecology* **101**, 1–11 (2020). The error has been corrected in the HTML and PDF versions of the Article.

