# Supplementary material for: Large-scale shift in the structure of a kelp forest ecosystem co-occurs with an epizootic and marine heatwave
Source: Commun Biol. 2021 Mar 5;4:298. doi: 10.1038/s42003-021-01827-6 (PMC7935997; doi:10.1038/s42003-021-01827-6)
Supplement: Supplementary file 2 — Supplementary Information [file 42003_2021_1827_MOESM2_ESM.pdf]

Supplementary Material

Supplementary Table 1 – Component-wise PLSR results for each date range (Figure 3).

	Full Timeseries	Preceding MHW	Recent Decade	With Biological Forcings
Date Range	1985 – 2016	1985 – 2013	2003 – 2016	2003 - 2016
Component 1 r^2	0.303	0.224	0.517	0.498

Supplementary Figure 1 – PLSR models and forecasts using physical drivers (a) and physical and biological drivers (b) overlaid on satellite derived kelp canopy. Forecasted scenarios use environmental variables (MEI, NPGO, PDO, Mean Hs, seasonal SST (spring and summer), MHW days, and seasonal NO_3 (spring and summer) conditions at the climatological mean for 2017 to 2019. For all other years (1985 - 2016 and 2003 - 2016), these variables are the environmentally derived indices.

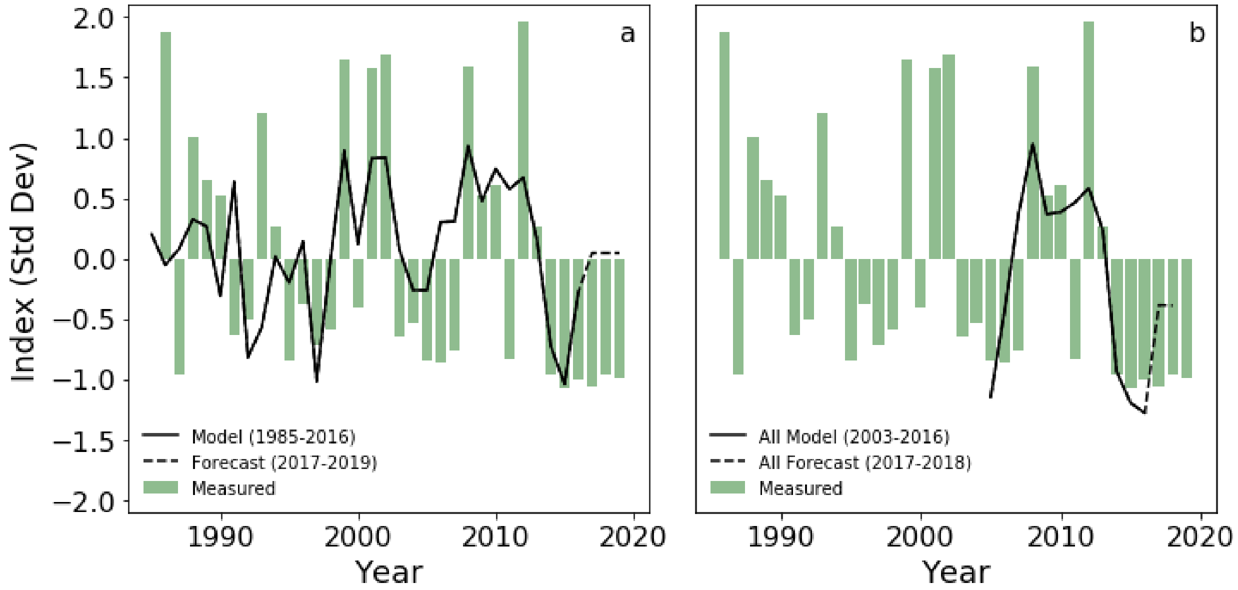

Supplementary Figure 2 – Least squares regression (LSR) fits for standardized indices of environmental ((a) bull kelp, (b) spring nitrate, and (d) MHW days) and biological ((c) sunflower star and (e) purple urchin) preceding the NE Pacific MHW and following the NE Pacific MHW. Date ranges depended on data availability for each variable. An ordinary LSR (OLSR) was applied to all variables except the sunflower star's preceding NE Pacific MHW date range (panel c; 2003 – 2013) where a second degree polynomial LSR was applied. For variable-wise regression statistics see S4. Shading around the regression lines represents the 95% confidence intervals.

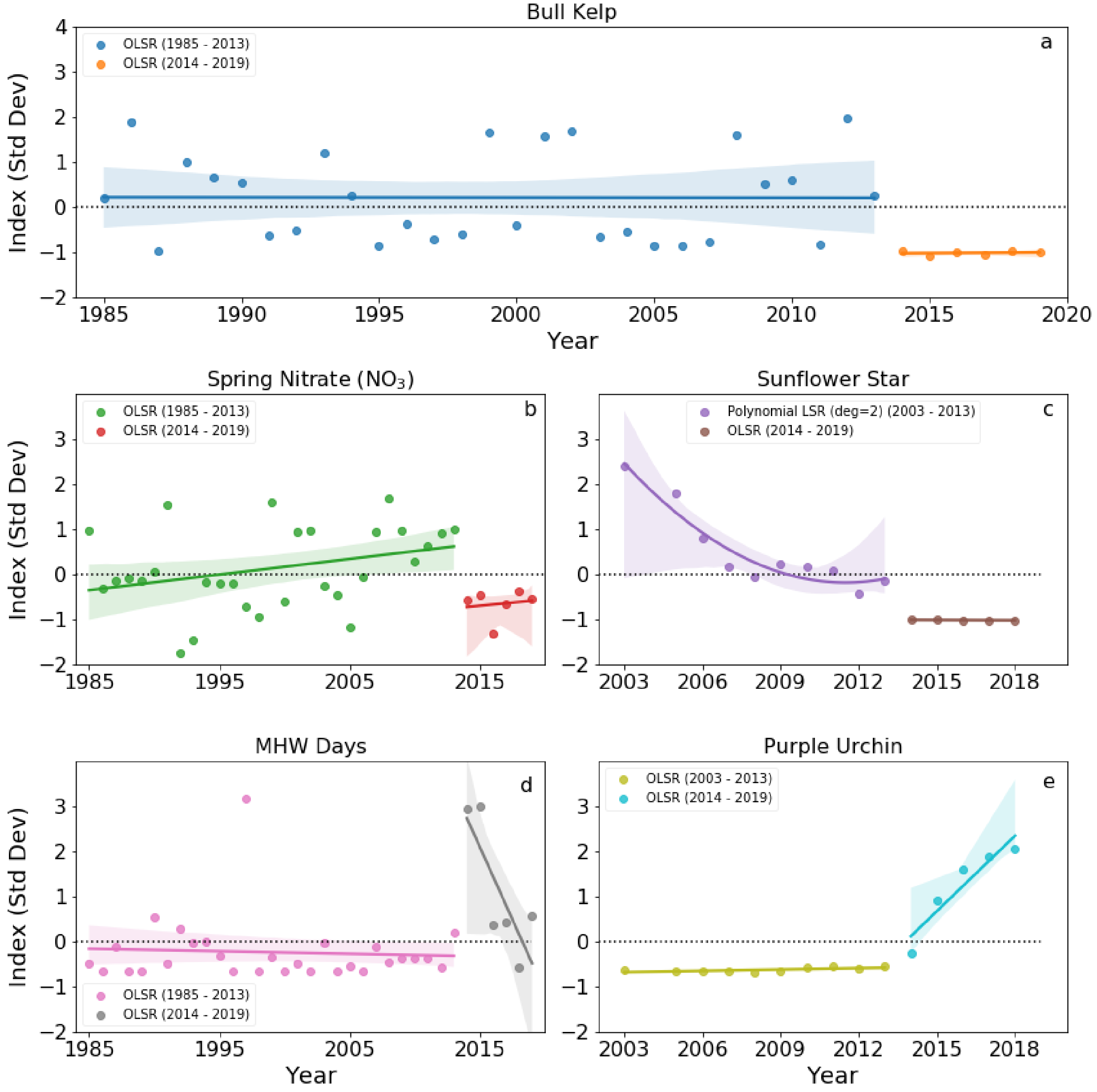

Supplementary Table 2 - Least squares regression (LSR) fits for standardized indices of environmental (bull kelp, spring nitrate, MHW days) and biological (purple urchin and sunflower star) show in Fig. 4 and S3. Date ranges presented for each timeframe (preceding MHW, following MHW, full timeseries) depended on data availability for each variable. Preceding the NE Pacific MHW, the date ranges of 1985 to 2013 and 2003 to 2013 were used for environmental and biological variables, respectively. Following the NE Pacific MHW, date ranges were from 2014 to 2019 and 2014 to 2018 were used for environmental and biological variables, respectively. Full timeseries date ranges were 1985 to 2019 for environmental variables, and 2003 to 2018 for biological variables. Bolded and grey highlighted cells designate statistically significant relationships (p < 0.05). Ordinary LSR was used for all trends presented below with the exception of the preceding MHW sunflower star trend, where a second order polynomial LSR was applied (indicated with *).

**Index**	**Preceding MHW** (1985 – 2013 or 2003 – 2013)			**Following MHW** (2014 – 2019 or 2014 -2018)			**Full Timeseries** (1985 – 2019 or 2003 to 2018)		
	slope	r^2	*p*-value	slope	r^2	*p*-value	slope	r^2	*p*-value
Bull kelp	$-4.6x10^{-4}$	$1.6x10^{-5}$	0.98	$3.7x10^{-3}$	$2.1x10^{-2}$	0.78	$-3.0x10^{-2}$	$9.2x10^{-2}$	$7.7x10^{-2}$
Spring nitrate	$3.5\ x10^{-2}$	0.11	$8.6x10^{-2}$	$2.8x10^{-2}$	0.024	0.77	$6.4x10^{-4}$	$5.4x10^{-5}$	0.97
MHW days	$-5.5x10^{-3}$	$4.2x10^{-3}$	0.74	**-0.64**	**0.66**	**$5.03x10^{-2}$**	$2.7x10^{-2}$	$7.4x10^{-2}$	0.11
Purple urchin	**$9.8x10^{-3}$**	**0.46**	**$3.03x10^{-2}$**	**0.56**	**0.88**	**$1.8x10^{-2}$**	**0.25**	**0.64**	**$3.1x10^{-4}$**
Sunflower star	***-0.21**	***0.84**	***$1.3x10^{-6}$**	$-2.1x10^{-2}$	0.72	$6.8x10^{-2}$	**-0.21**	**0.84**	**$1.3x10^{-6}$**

Supplementary Table 3 – Index data sources for all environmental (largescale and local-scale) and biological indices. Detailed descriptions of large and local-scale forcings and their influences on kelp dynamics are listed below the table.

Response Variable	Predictor Variables		
1985 – 2019			2003 - 2018
Kelp Index	Largescale Indices	Local-scale Indices	Biological Indices
**USGS Landsat derived canopy area** - https://earthexplorer.usgs.gov	**NPGO** - http://www.o3d.org/npgo/	**SST** - https://www.ncei.noaa.gov/erddap/griddap/ncdc_oisst_v2_avhrr_by_time_zlev_lat_lon.html	**Purple urchin and Sunflower star densities** - http://data.reefcheck.us/[1] and California Department of Fish and Wildlife (L.R.B)
	**PDO** - http://research.jisao.washington.edu/pdo/	**MHW Days** - Hobday et al. 2016[2]	
	**MEI** - https://www.esrl.noaa.gov/psd/enso/mei/	**[NO_3]** - Garcia-Reyes et al. 2014[3]	
		**H_s** - https://www.ndbc.noaa.gov/ - station 46013	

SST Index –SST conditions effect the distribution (physiological temperature threshold), gametophyte maturation[4], and the seasonal growth rates[5].

NO_3 Index – Nitrate conditions fuel growth seasonally. Growth rates are primarily high in the spring and early summer due to the availability of nutrient rich water brought to the surface by seasonal upwelling. Growth rates are generally low in the summer due to limited nitrate conditions [5,6].

H_s Index – Bull kelp are an annual algal species and in exposed regions, such as the northern California coast, are typically removed by strong wave forces during fall and winter storms. Therefore, seasonal and annual trends in significant wave height influence canopy distribution [6].

MEI Index – the Multivariate El Niño/Southern Oscillation (ENSO) Index (MEI.v2) is indicative of global climate disruptions and derived from five different variables (sea level pressure, sea surface temperature, zonal and meridional components of the surface wind, and outgoing longwave radiation). Disruptions to oceanographic conditions via ENSO patterns influence SST, NO_3, and wave height conditions (H_s). Studies have found ENSO to be an important driver of kelp dynamics across the globe[7–11].

NPGO Index – the North Pacific Gyre Oscillation is an oceanic climate index derived from the second mode of sea surface height variability in the northeast Pacific and influences sea surface nutrient dynamics in the North Pacific Gyre and California Current. Many studies in the NE Pacific have found NPGO to be an important driver of regional kelp dynamics[10,12,13].

PDO Index – the Pacific Decadal Oscillation index is derived from the first mode of sea surface temperature variability in the north Pacific poleward of 20°N. Many studies in the NE Pacific have found PDO to be an important driver of regional kelp dynamics[10,12,13].

Supplementary Figure 3 – Box and whisker plot for all predictor (environmental and biological) and response (kelp canopy) variables. Variable-wise outliers are defined as datapoints outside 1.5 times the interquartile range (1.5*IQR; black points). Total sample number across the entire variable timeseries is represented by n_t. Sampling frequency (annual, monthly, daily, or hourly) is depicted by the grey boxes near the x-axis.

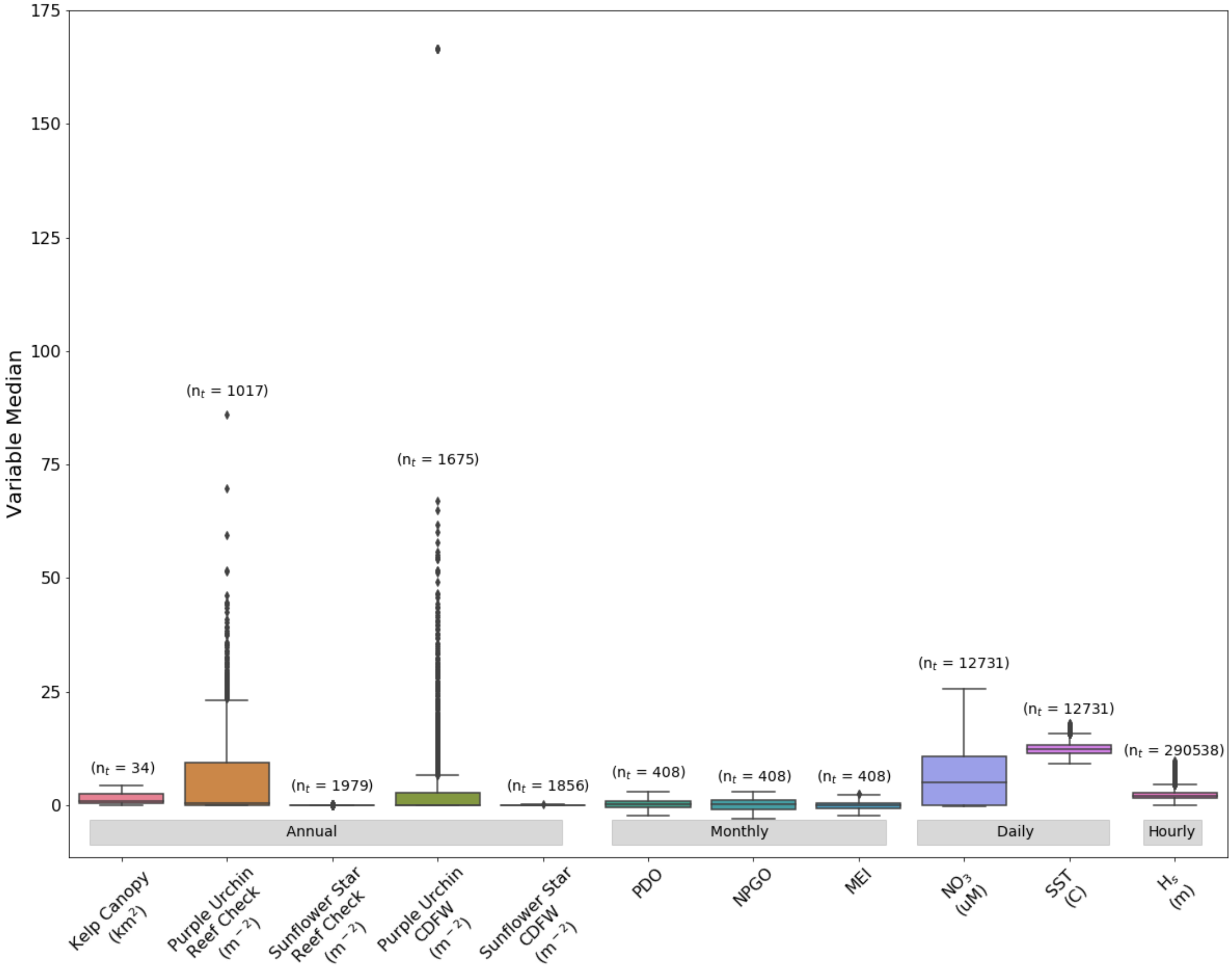

Supplementary Figure 4 – Temporal (a) and spatial (b and c) representation of sub-tidal sampling efforts in Sonoma and Mendocino Counties in the northern California, USA region between 2003 and 2018 by California Department of Fish and Wildlife (CDFW) and Reef Check California.

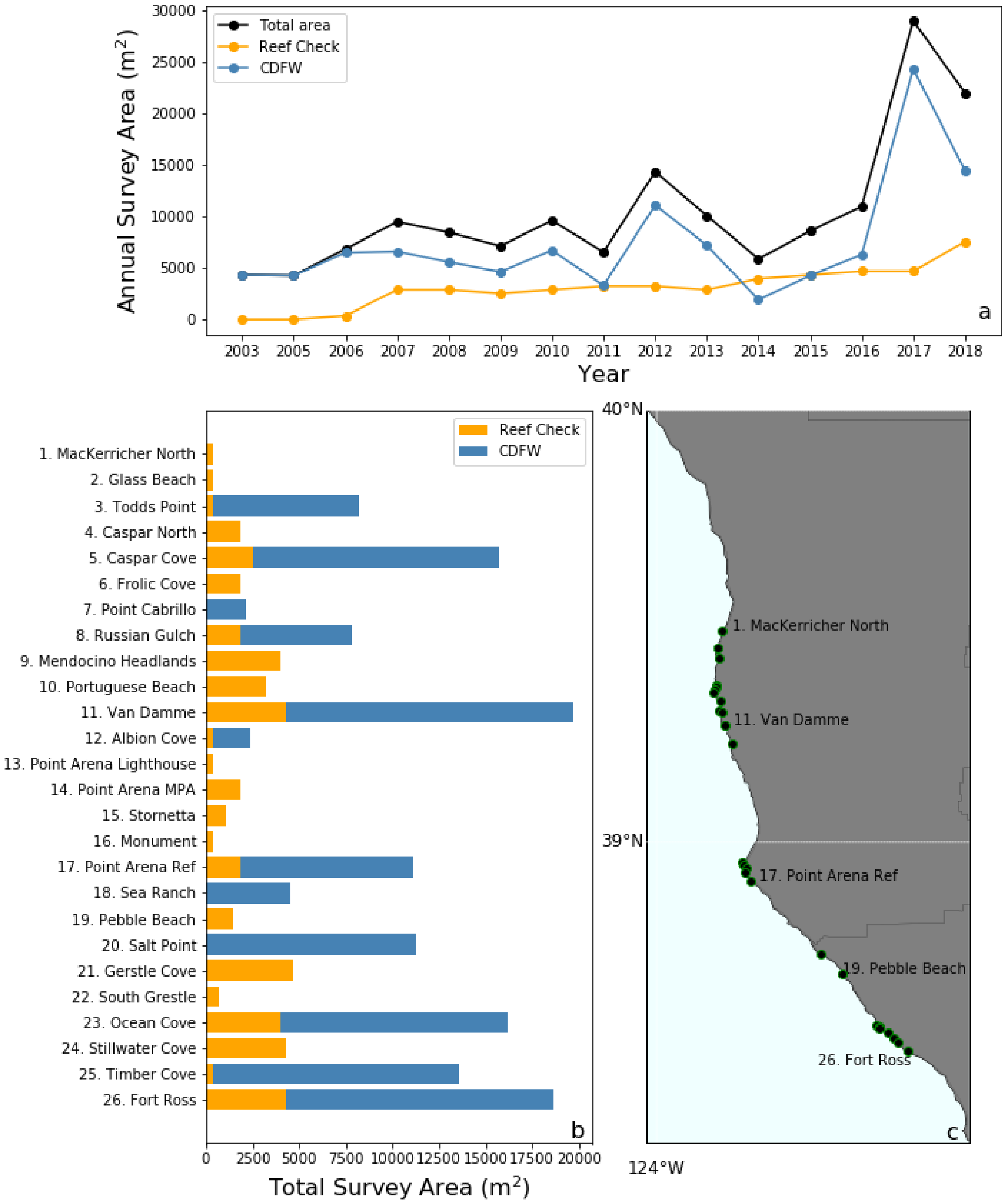

Supplementary Figure 5 – Correlation matrix of all environmental and biological variables used in the partial least squares regression (PLSR) analysis. The upper panel corner shows the scatter plots Pearson correlation coefficients (r) for each pair-wise relationship. The lower corner shows the kernel density distribution for each pair-wise relationship. The diagonal shows the data distribution for each variable. Strong co-linearity exists between seasonal sea surface temperature (SST) and nitrate (NO_3) conditions.

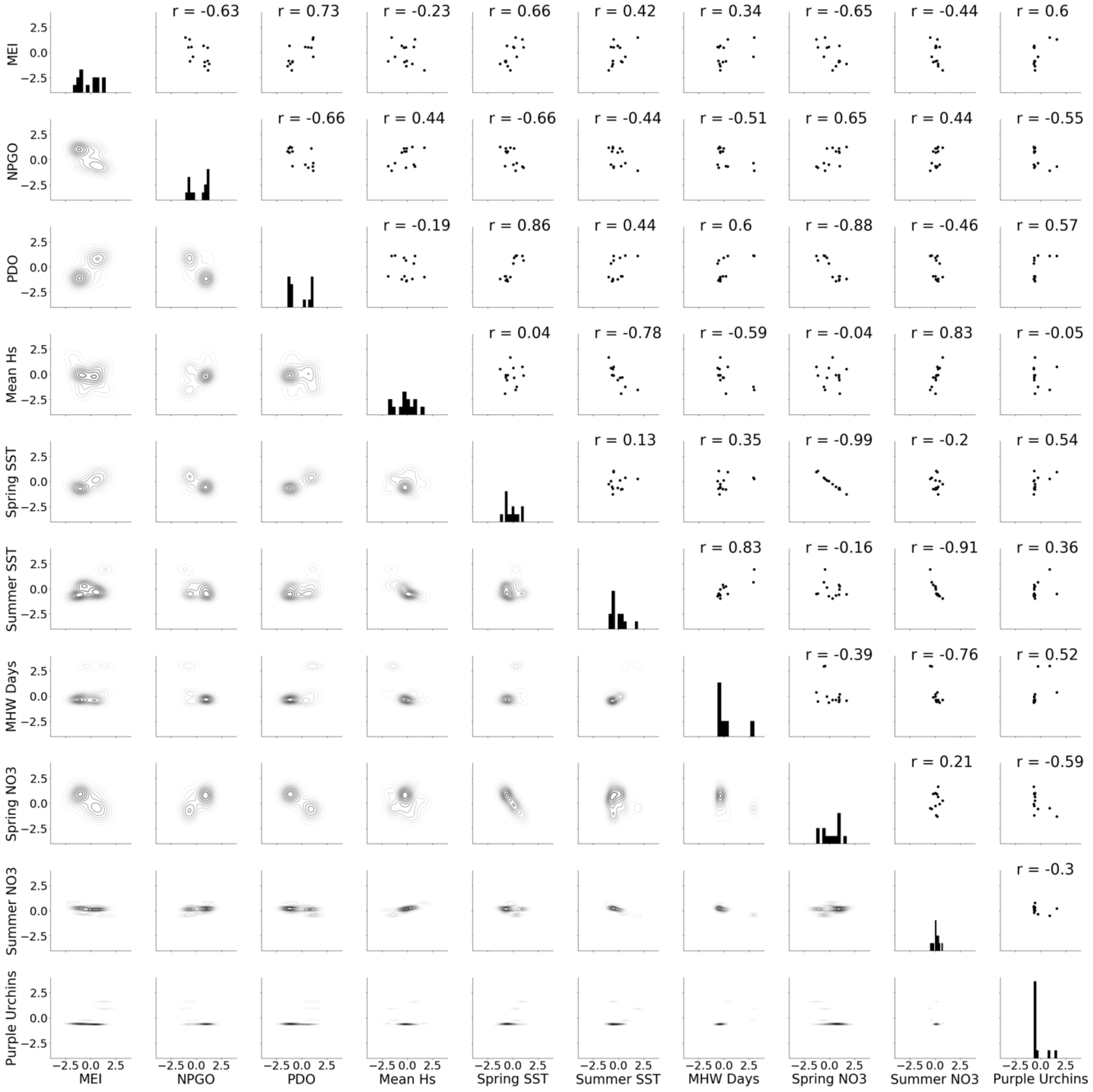

Supplementary Figure 6 – Partial least squares regression (PLSR) component- and variable-wise cross-validation results presented as the mean squared error (MSE) for (a) environmental indices (1985 – 2016) and (b) environmental and purple urchins (all indices; 2003 – 2016).

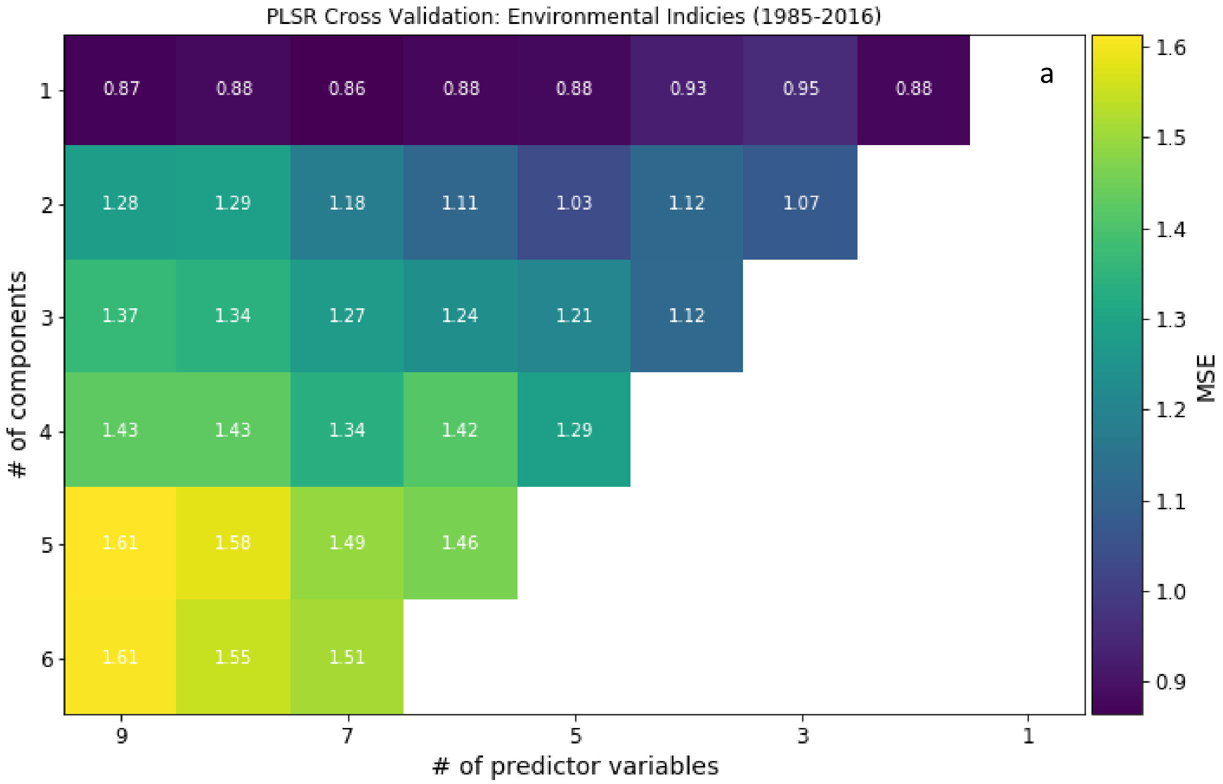

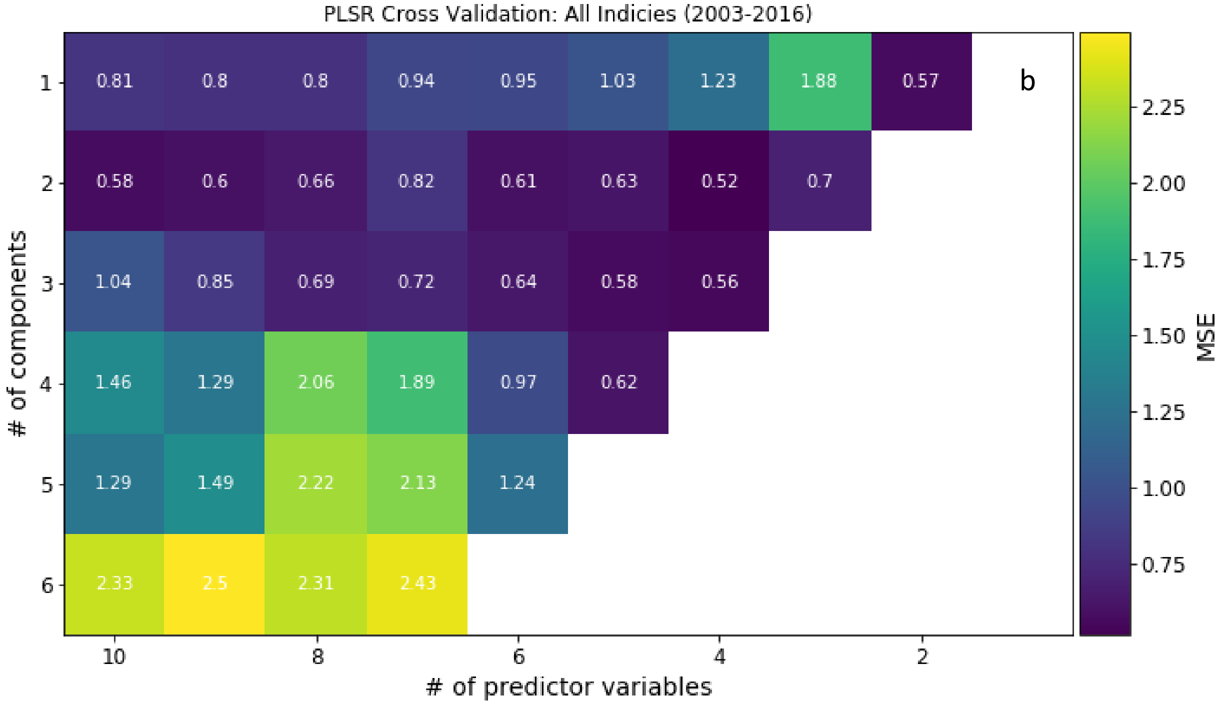

Supplementary References

1. McHugh, T., Abbott, D. & Freiwald, J. Phase shift from kelp forest to urchin barren along California's North Coast. in *Western Society of Naturalists* (2018).
2. Hobday, A. J. *et al.* A hierarchical approach to defining marine heatwaves. *Prog. Oceanogr.* **141**, 227–238 (2016).
3. García-reyes, M., Largier, J. L. & Sydeman, W. J. Progress in Oceanography Synoptic-scale upwelling indices and predictions of phyto- and zooplankton populations. *Prog. Oceanogr.* **120**, 177–188 (2014).
4. Vadas, R. L. Ecological implications of culture studies on nereocystis luetkeana. *J. Phycol.* **8**, 196–203 (1972).
5. Wheeler, W. N., Smith, R. G. & Srivastava, L. M. Seasonal Photosynthetic Performance of Nereocystis-Luetkeana. *Can. J. Bot. Can. Bot.* **62**, 664–670 (1984).
6. Springer, Y., Hays, C., Carr, M., Mackey, M. & Bloeser, J. Ecology and management of the bull kelp, Nereocystis luetkeana: A synthesis with recommendations for future research. 48 (2006).
7. Tegner, M. J. M. & Dayton, P. K. P. El Nino effects on southern California kelp forest communities. *Adv. Ecol. Res.* **17**, 243–279 (1987).
8. Friedlander, A. M. *et al.* Kelp forests at the end of the earth : 45 years later. *PLoS One* **15**, 1–23 (2020).
9. Tally, L. ., Pickard, G. L., Emery, W. J. & Swift, J. H. *Descriptive Physical Oceanography: An Introduction*. (Elsevier Ltd., 2011).
10. Cavanaugh, K. C., Siegel, D. A., Reed, D. C. & Dennison, P. E. Environmental controls of giant-kelp biomass in the Santa Barbara Channel, California. *Mar. Ecol. Prog. Ser.* **429**, 1–17 (2011).
11. Schiel, D. R. & Foster, M. S. *The Biology and Ecology of Giant Kelp Forests*. (2015).
12. Hamilton, S. L., Caselle, J. E. & Hamilton, S. L. Exploitation and recovery of a sea urchin predator has implications for the resilience of southern California kelp forests. *Proc. R. Soc. B* **282**, (2014).
13. Pfister, C. A., Berry, H. D. & Mumford, T. The dynamics of Kelp Forests in the Northeast Pacific Ocean and the relationship with environmental drivers. *J. Ecol.* 1–14 (2017). doi:10.1111/1365-2745.12908
