# Supplementary material for: Large-scale shift in the structure of a kelp forest ecosystem co-occurs with an epizootic and marine heatwave
Source: Commun Biol. 2021 Mar 5;4:298. doi: 10.1038/s42003-021-01827-6 (PMC7935997; doi:10.1038/s42003-021-01827-6)
Supplement: Supplementary file 3 — Description of Additional Supplementary Files [file 42003_2021_1827_MOESM3_ESM.pdf]

Description of Additional Supplementary Files

File name: Supplementary Data

File Description: Source data for Figures 1-4
