# Supplementary material for: Large-scale shift in the structure of a kelp forest ecosystem co-occurs with an epizootic and marine heatwave
Source: Commun Biol. 2021 Mar 5;4:298. doi: 10.1038/s42003-021-01827-6 (PMC7935997; doi:10.1038/s42003-021-01827-6)
Supplement: Supplementary file 5 — Reporting Summary [file 42003_2021_1827_MOESM5_ESM.pdf]

nature research

Corresponding author(s): Meredith L. McPherson
Raphael M. Kudela

Last updated by author(s): Jan 25, 2021

Reporting Summary

Nature Research wishes to improve the reproducibility of the work that we publish. This form provides structure for consistency and transparency in reporting. For further information on Nature Research policies, see our Editorial Policies and the Editorial Policy Checklist.

Statistics

For all statistical analyses, confirm that the following items are present in the figure legend, table legend, main text, or Methods section.

n/a	Confirmed	
☐	☒	The exact sample size (*n*) for each experimental group/condition, given as a discrete number and unit of measurement
☐	☒	A statement on whether measurements were taken from distinct samples or whether the same sample was measured repeatedly
☐	☒	The statistical test(s) used AND whether they are one- or two-sided<br>*Only common tests should be described solely by name; describe more complex techniques in the Methods section.*
☐	☒	A description of all covariates tested
☐	☒	A description of any assumptions or corrections, such as tests of normality and adjustment for multiple comparisons
☐	☒	A full description of the statistical parameters including central tendency (e.g. means) or other basic estimates (e.g. regression coefficient) AND variation (e.g. standard deviation) or associated estimates of uncertainty (e.g. confidence intervals)
☐	☒	For null hypothesis testing, the test statistic (e.g. F, t, r) with confidence intervals, effect sizes, degrees of freedom and P value noted<br>*Give P values as exact values whenever suitable.*
☒	☐	For Bayesian analysis, information on the choice of priors and Markov chain Monte Carlo settings
☒	☐	For hierarchical and complex designs, identification of the appropriate level for tests and full reporting of outcomes
☐	☒	Estimates of effect sizes (e.g. Cohen's *d*, Pearson's *r*), indicating how they were calculated

Our web collection on statistics for biologists contains articles on many of the points above.

Software and code

Policy information about availability of computer code

Data collection	All data collected and used for this analysis is publicly available online or via requests from responsible organizations. See supplemental material (S4) for specific links and organizations where data was acquired.
Data analysis	Commercial software (L3Harris Geospatial ENVI, ver. 5.5) was used for image processing prior to data analysis.<br>Data analysis and visualization was conducted in Python 3.7 using custom written code and utilized available Python modules (GDAL, sklearn, pandas, numpy, scipy, matplotlib, etc.).

For manuscripts utilizing custom algorithms or software that are central to the research but not yet described in published literature, software must be made available to editors and reviewers. We strongly encourage code deposition in a community repository (e.g. GitHub). See the Nature Research guidelines for submitting code & software for further information.

Data

Policy information about availability of data

All manuscripts must include a data availability statement. This statement should provide the following information, where applicable:
- Accession codes, unique identifiers, or web links for publicly available datasets
- A list of figures that have associated raw data
- A description of any restrictions on data availability

The data that support the findings of this study are available from the authors on reasonable request, see supplemental material for specific data sets used.

Field-specific reporting

Please select the one below that is the best fit for your research. If you are not sure, read the appropriate sections before making your selection.

☐ Life sciences ☐ Behavioural & social sciences ☒ Ecological, evolutionary & environmental sciences

For a reference copy of the document with all sections, see nature.com/documents/nr-reporting-summary-flat.pdf

Ecological, evolutionary & environmental sciences study design

All studies must disclose on these points even when the disclosure is negative.

Study description

This study aimed to determine the influence of large-scale, local-scale, and biological factors on northern California kelp canopy coverage using a 34 year timeseries.
Treatment factors included:
The large-scale forcings included the Multivariate El Niño/Southern Oscillation Index (MEI), North Pacific Gyre Oscillation (NPGO), and Pacific Decadal Oscillation (PDO). Local-scale forcings included significant wave height (Hs), as well as multiple signatures corresponding with coastal upwelling, including upwelling index (UI), sea surface temperature (SST), and surface nitrate concentrations (NO3) driven by SST conditions. Marine heatwave days (MHW) and sea surface nitrate concentrations were calculated based on published methodology from Hobday et al. and Garcia-Reyes et al., respectively. Biological forcings included purple sea urchin (Strongylocentrotus purpuratus) and sunflower star (Pycnopodia helianthoides) densities obtained from California Department of Fish and Wildlife (CDFW) and Reef Check California. Indices were corrected for climatology and standardized to each variable mean and standard deviation.
This approach resulted in a single annual value (or seasonal) for each predictor and response factor (n=34). No replicates were used.

Research sample

Kelp are distributed along temperate rocky coastlines in upwelling systems, but in the north eastern pacific region, bull kelp ranges from Baja California to Alaska. The primary subject of this study is bull kelp (Nereocystis luetkeana; Order: Laminariales), which has experienced significant reduction in northern California over the last 5 years, and provided impetus for this study. Futhermore, there is evidence that climate change and other anthropogenic factors are contributing to declines in kelp forests globally.

Sampling strategy

34 years of satellite derived imagery is available via USGS Landsat series. Imagery is collected every 16 days (regardless of atmospheric conditions), however only cloud-free or nearly cloud-free imagery can be used for analysis. In addition, the seasonal cycle of bull kelp limits maximum canopy expression to a short window during late summer, early fall. We chose to use a single image for each year that approximately represents maximum canopy distribution for that year. Imagery selection was between August 1 and Oct. 31. The environmental indices, which are sometimes available at higher temporal frequency, was further broken down into annual indices. Our approach is as follows:

For physical indices (1985 to 2018) that were measured at hourly (Hs) and daily frequency (SST, NO3), data were temporally binned into monthly averages across 1985 to 2018. For all physical indices, climatology was determined by removing the long-term monthly mean from data at a monthly frequency. To scale monthly indices to the annual frequency of the kelp index, the monthly climatologically corrected indices were averaged to annual or seasonal values (e.g. summer SST, spring NO3, winter Hs). For biological data obtained from Reef Check California (2007 to 2018) and (2003 to 2018) measured at an annual frequency, climatology was determined by removing the long-term mean from each annual measurement. Since biological data was collected during summer months when kelp biomass is at its peak, the influence of seasonal or inter-annual variation in biological data was not investigated.

Data collection

All data was downloaded and processed from the sources in supplemental material (S4) by Meredith L. McPherson.

Timing and spatial scale

Data was collected between 1985 and 2019. Statistical analyses were conducted for 1985-2019 and 2003 to 2018.
These windows were chosen specifically because the satellite derived canopy coverage from Landsat 30m spatial resolution imagery was available between 1985 and 2019. All other physical data was available overlapping the Landsat products. Biological data was only available between 2003 - 2018. This is made clear in the description of methods and results in the manuscript.

The total study area is across 350 km of coastal waters on the northern California, USA coastline. Each Landsat pixel represents 900 m^2 area at the finest spatial resolution (not presented in the manuscript) and is the native resolution used in the analysis. Kelp canopy data are presented at courser resolutions in the manuscript, including the total area in the entire northern California region and in 90 m (3 Landsat pixels) meridional space.

Data exclusions

No data were excluded from the analysis.

Reproducibility

Reproducibility is not relevant to this study because the focus was statistical analysis of environmental predictor variables on a single predictend variable across a timeseries.

Randomization

Environmental variables were divided into two groups, physical and biological depending on the larger and local scale processes (ocean and atmospheric forcing vs. grazer/predator niche) and the role of that variable on the kelp forest ecosystem. Covariates, of which many of our environmental variables are, were controlled using partial least squares regression analysis where scores and weights describe the covariance across predictor variables.

Blinding

Blinding was not conducted during analysis because it was not necessary to remove bias while conducting this particular statistical approach.

Did the study involve field work? ☐ Yes ☒ No

Reporting for specific materials, systems and methods

We require information from authors about some types of materials, experimental systems and methods used in many studies. Here, indicate whether each material, system or method listed is relevant to your study. If you are not sure if a list item applies to your research, read the appropriate section before selecting a response.

Materials & experimental systems

n/a	Involved in the study
☒	☐ Antibodies
☒	☐ Eukaryotic cell lines
☒	☐ Palaeontology and archaeology
☒	☐ Animals and other organisms
☒	☐ Human research participants
☒	☐ Clinical data
☒	☐ Dual use research of concern

Methods

n/a	Involved in the study
☒	☐ ChIP-seq
☒	☐ Flow cytometry
☒	☐ MRI-based neuroimaging
